# The Association Between Depressive Symptoms and Accumulation of Stress Among Black Men in the Health and Retirement Study

**DOI:** 10.1093/geroni/igaa047

**Published:** 2020-09-29

**Authors:** Roland J Thorpe, Ryon Cobb, Keyonna King, Marino A Bruce, Paul Archibald, Harlan P Jones, Keith C Norris, Keith E Whitfield, Darrell Hudson

**Affiliations:** 1 Program for Research on Men’s Health, Hopkins Center for Health Disparities Solutions, Johns Hopkins Bloomberg School of Public Health, Baltimore, Maryland; 2 Department of Sociology, University of Georgia, Athens; 3 Department of Health Promotion, University of Nebraska Medical Center, Omaha; 4 Department of Population Health Science, John D. Bower School of Population Health, University of Mississippi Medical Center, Jackson; 5 Department of Social Work, College of Staten Island, The City University of New York; 6 Department of Microbiology, Immunology and Genetics, University of North Texas Health Science Center, Fort Worth; 7 Department of Medicine, University of California, Los Angeles; 8 Department of Psychology, University of Nevada Las Vegas; 9 Brown School at Washington University in St. Louis, Missouri

**Keywords:** Allostatic load, Black men, Depressive symptoms, Inequities, Men, Men’s health, Stress

## Abstract

**Background and Objectives:**

Among the multiple factors posited to drive the health inequities that black men experience, the fundamental role of stress in the production of poor health is a key component. Allostatic load (AL) is considered to be a byproduct of stressors related to cumulative disadvantage. Exposure to chronic stress is associated with poorer mental health including depressive symptoms. Few studies have investigated how AL contributes to depressive symptoms among black men. The purpose of the cross-sectional study was to examine the association between AL and depressive symptoms among middle- to old age black men.

**Research Design and Methods:**

This project used the 2010 and 2012 wave of the Health and Retirement Study enhanced face-to-face interview that included a biomarker assessment and psychosocial questionnaire. Depressive symptoms, assessed by the endorsement of 3 or more symptoms on the Center for Epidemiological Studies—Depression 8-item scale, was the outcome variable. The main independent variable, AL, score was calculated by summing the number values that were in the high range for that particular biomarker value scores ranging from 0 to 7. black men whose AL score was 3 or greater were considered to be in the high AL group. Modified Poisson regression was used to estimate prevalence ratios (PRs) and corresponding 95% confidence intervals (CIs).

**Results:**

There was a larger proportion of black men in the high AL group who reported depressive symptoms (30.0% vs. 20.0%) compared with black men in the low AL group. After adjusting for age, education, income, drinking, and smoking status, the prevalence of reporting 3 or more depressive symptoms was statistically significant among black men in the high AL group (PR = 1.61 [95% CI: 1.20–2.17]) than black men in the low AL group.

**Discussion and Implications:**

Exposure to chronic stress is related to reporting 3 or more depressive symptoms among black men after controlling for potential confounders. Improving the social and economic conditions for which black men work, play, and pray is key to reducing stress, thereby potentially leading to the reporting of fewer depressive symptoms.

**Translational Significance:** There is a lack of research focusing on depressive symptoms and how adaptations are made to a chronic stressor as measured by physiological outcomes like allostatic load among middle- to old age black men. Our work demonstrates the physiological adaptation to stress may be linked to the reporting of three or more depressive symptoms among middle- to old age black men. This work underscores the importance of fostering of protective coping strategies or strengthening resilience for middle- to old age black men to test if such strategies can reduce allostatic load and improve health outcomes.

Stress is an environmental, social, or internal demand that activates physiological, psychological, or behavioral response (Cohen et al., 1995; Holmes & Rahe, 1967; Thoits, 1995). Stress is considered a major contributor in the development of poor mental health conditions, including depression and depressive symptoms (Berger & Sarnyai, 2015; Dohrenwend et al., 1992; Mezuk et al., 2013; Turner & Lloyd, 1999). Chronic activation of the stress response system can lead to dysregulated immune, hormonal, and cardiovascular functioning in a manner that is deleterious to health (Geronimus et al., 2006; McEwen, 2003; McEwen & Wingfield, 2003). Chronic stress can also disrupt psychological functioning as it is generally thought to lead to the development of poor mental health conditions, including depression (Berger & Sarnyai, 2015; Dohrenwend et al., 1992; Mezuk et al., 2013; Turner & Lloyd, 1999). There is a growing body of research that has investigated the influence stress or stressors impact on the health and well-being of black men across the life course (Brown & Hargrove, 2017; Thorpe et al., 2015). Findings from these studies indicate that black men experience a substantial amount of stress across the life course, including lower socioeconomic status and exposure to racism, compared to white men (Brown & Hargrove, 2017; Thorpe et al., 2015). Exposure to stress over the life course could affect the experience of depressive symptoms among middle- to old age black men.

Scholars have developed several theoretical frameworks to explain the effects of stress on health, particularly how chronic stress “gets under the skin” to affect health (Krieger, 2001). McEwen and Wingfield (2003) described allostatic load as the “wear and tear” on the body that occurs and is exacerbated with greater exposure to chronic stress. As a result of higher allostatic load, Geronimus et al. (2006) theorize that the weathering occurs in which people under chronic stress experience accumulated disadvantage over the life course leading to accelerated aging evidenced by earlier onset of health conditions.

In addition to weathering, the connection among social context, stress, coping practices, and mental health has also been explored by researchers. Jackson et al. (2010) described the Environmental Affordances Model in which it is hypothesized that, in response to stress, people cope with the resources that are most proximal and socially acceptable in their environments. Because of high levels of racial residential segregation throughout the United States, predominately black communities have greater stress exposure attributable to lower levels of stable employment opportunities and adequate housing, and increases in community-level violence while simultaneously providing coping resources that are deleterious to health such as greater access to high-fat, calorie-dense food (comfort food) as well as alcohol and illicit drugs (Mezuk et al., 2010). According to this model, the practice of poor health behaviors (e.g., poor diet, overeating, and alcohol use) could provide relief from chronic stress and protect against the development of depression and other mental disorders. Yet, these poor health behaviors may simultaneously increase the risk of obesity and subsequent chronic diseases (Jackson et al., 2010; Mezuk et al., 2013). Hudson et al. (2016) found that black men reported the use of unhealthy behaviors, particularly excessive alcohol consumption and illicit drug use, to cope with stress. These patterns of coping have many different effects across the life course ranging from black men’s ability to attain education and employment opportunities to incarceration as well as physical health problems. These theoretical frameworks are helpful in the examination of depressive symptoms among middle- to old age black men.

The challenges associated with understanding depression among men are exacerbated with racial considerations as the combination of hegemonic norms about race and gender makes it more difficult to discern depressive symptoms among black men (Keating, 2009; McGuire & Miranda, 2008; Metzl, 2013; Whaley, 2001). Scholars have noted that behavioral health providers have historically been more likely to diagnose black men with schizophrenia than depression (Metzl, 2009; Whaley, 2001). Furthermore, Bryant-Bedell and Waite (2010) found that black men experienced depression in the past but did not realize they were depressed. Participants in their study described feelings of depression, including irritability, isolation, and depressed mood (Bryant-Bedell & Waite, 2010). Access to mental health care is a significant challenge for black men as they are among the most medically underserved groups in the United States (Hudson et al., 2016; Snowden et al., 2009; Snowden & Yamada, 2005; Thorpe, Bowie et al., 2013). In a focus groups study with low-income black men, Hudson et al. (2016) uncovered significant barriers to behavioral health care, including affordability, stigma, mistrust, and masculinity-related social norms. Men in this study reported that they did not want to seek regular medical treatment in general due to concerns about the affordability of treatment, especially if they did not have health insurance or adequate financial resources.

Even when mental health conditions are detected, especially in primary care settings where most mental health treatment occurs in the United States, these conditions are often unrecognized or poorly treated (Conwell et al., 2011; Hudson et al., 2013). Mays et al. (2018) found that among black men who did meet the criteria for mood disorders, they were more likely to experience greater chronicity and have low levels of mental health service utilization. In the context of primary care, it is likely that many older men are not identified as having a mental health issue when they seek medical treatment in primary care settings and because this is where most Americans are diagnosed with depression, they may never be seen by mental health specialty providers (González et al., 2010; Miranda & Cooper, 2004; Nutting et al., 2008).

Black men are exposed to stress from multiple sources throughout their lives and the number and chronicity of these exposures can have implications for psychological outcomes during middle- and late life (Bruce & Thorpe, 2019; Thorpe & Archibald, 2019). Data from existing psychiatric epidemiologic studies indicate that black men have lower rates of depression compared to black women and white men (Williams et al., 2007). However, large epidemiologic studies that focus on mental health outcomes that include black men tend to exclude important groups such as older men (Reynolds et al., 2015), men living in under-resourced rural or inner-city areas (Metzl, 2013; U.S. Department of Health and Human Services, 2001), or institutionalized men (Jackson et al., 2004). This lack of representation can be linked to the difficultly to recruit black men to participate in research (Barrett et al., 2017; Webb et al., 2019).

Jackson et al. (2010) have argued that people cope with “resources” that are accessible, affordable, and socially acceptable in their neighborhoods. Jackson et al. posit that consuming “comfort food,” alcohol, or illicit drugs may provide temporary stress relief and serve as a buffer against the onset of depression and other mental disorders. Yet, these poor health behaviors may simultaneously increase the risk of obesity and subsequent chronic diseases (Jackson et al., 2010; Mezuk et al., 2013). Mezuk et al. (2010) presented data indicating that stress exposure was related to an increase in unhealthy behaviors (e.g., smoking and consuming alcohol) and lower risk of depression. These results suggest that exposure to stress along with the employment of stress coping strategies likely increases allostatic load, undermining the mental and physical health of black men as they age. In addition, the development of social contexts where black men have few safe and healthy coping options is important (LaVeist et al., 2011; Thorpe, Bowie et al., 2013; Thorpe, Wilson-Frederick et al., 2013).

## Study Rationale

Although depressive symptoms, such as fatigue, anhedonia, and feelings of sadness, are associated with psychosocial stress (Lincoln et al., 2010, 2011; Woodward et al., 2013), little is known about depressive symptoms and stress among middle- to old age black men (Woodward et al., 2011). Furthermore, data specific to black men, especially middle- and older-age black men, are scarce (Woodward et al., 2011). There is evidence that black men are severely medically underserved, even more regarding their mental health (Satcher, 2008). Lack of data, including service utilization, treatment preferences, and symptom manifestation across race/ethnicity among middle- to old age black men means that efforts to address mental health among men are likely underpowered and may not align well with these populations. However, the ramifications are palpable as the effects of stress and coping responses could accumulate across the life course and affect the quality of life and overall health of men as they age. This could be especially true for black men who reside in resource-poor settings and rely on coping behaviors that are deleterious, such as consumptions of alcohol, tobacco, or illicit drugs to mitigate the effects of chronic stress exposure (Jackson et al., 2010). The purpose of the study was to examine the association between stress as measured by allostatic load and depressive symptoms among middle- to old age black men.

## Methods

### Data and Sample

The Health and Retirement Study (HRS) is an ongoing longitudinal panel study that is representative of adults in the United States who are 50 years of age and older. HRS began data collection in 1992. However, in 2006, a random half sample of HRS households was selected to receive an enhanced face-to-face (EFTF) interview, blood-based biomarker, and anthropometric assessments (Crimmins et al., 2013). The remaining half sample of the 2006 sample received the EFTF interviews, blood-based biomarker, and physical assessments in 2008. These blood-based and anthropometric assessments are repeated on these individuals who participate 4 years later (i.e., 2010/2012). The present cross-sectional study included 850 black men aged 50–101 who completed the 2010 and 2012 biomarker and anthropometric assessments.

### Depressive Symptoms

Our depressive symptoms measure consists of the eight-item version of the Center for Epidemiological Studies-Depression scale (Karim et al., 2015; Turvey et al., 1999). Participants reported yes or no to whether they felt “much of the time during the past week”: (1) depressed, (2) everything was an effort, (3) that sleep was restless, (4) happy, (5) lonely, (6) that they enjoyed life, (7) sad, and (8) unmotivated and could not get going. The number of yes responses to items 1, 2, 3, 5, 7, 8 and the number of no responses to items 4 and 6 were summed to generate a summary depressive symptom score ranging from 0 to 8 (Steffick, 2000). Higher scores indicate more depressive symptoms. Consistent with previous work (Dang et al., 2020; Steffick, 2000), a binary variable was used to identify those middle- to old age black men who reported three or more depressive symptoms relative to those middle- to old age black men who reported fewer than three depressive symptoms.

### Allostatic Load

Seven biomarkers were used to measure allostatic load: glycosylated hemoglobin (HbA1c), C-reactive protein (CRP), high-density lipoprotein (HDL) cholesterol, total cholesterol (TC), waist circumference, resting heart rate, and Cystatin-C. Blood-based biomarkers included HbA1c, CRP, HDL, TC, and Cystatin-C. A trained HRS interviewer collected each respondent’s blood using the dried blood spot (DBS) method and shipped to a designated facility for processing. Additional details regarding the DBS methodology are described elsewhere (Crimmins et al., 2013; McDade et al., 2007). Waist circumference, blood pressure, and heart rate were measured separately along with other anthropometric data. Waist circumference was measured by wrapping a tape measure around the respondent’s waist as they stood and raised their arms. Heart rate was collected simultaneously as respondents sat down with both feet on the floor using an automated blood pressure monitor with approximately 45–60 s between each measurement. Three separate measures were taken and averaged to determine blood pressure and heart rate. Additional details regarding the protocol, special instructions, and other criteria required to properly measure each anthropometric indicator are provided in Crimmins et al. (2008).

An allostatic load score was created by identifying participants with values in the high-risk quartile: ≤25th percentile for HDL and ≥75th percentile for all of the biomarkers (Crimmins, 2003; Geronimus et al., 2006; Seeman et al., 2001; Suh et al., 2019). Participants received 1 point toward their allostatic load score for each biomarker in the high-risk quartile with a score ranging from 0 to 7. Allostatic load score was calculated by summing the number of values that score in the high-risk category. Participants whose allostatic load score was 3 or more were assigned to the high allostatic load group (Geronimus et al., 2006; Seeman et al., 1997, 2001).

### Other Covariates

The present study controlled for demographic, socioeconomic, and health behavioral factors that could be associated with high allostatic load and/or major depressive symptoms among middle- to old age black men. Age is a continuous variable of respondents’ reported age at the time of the interview. Education is classified as less than high school, high school graduate/GED, some college, and college and above. Income is based on monthly household income. Based on prior work and because of the skewness of the income variable, we took the natural log of income. Health behaviors included current smoking status dichotomized as current smoker and nonsmoker. Drinking status was based on the number of days per week the respondent consumes alcoholic beverages.

### Statistical Analysis

Wald tests and Pearson chi-squared tests were used to examine mean and proportional differences by allostatic load group for demographic factors, socioeconomic variables, and health behavioral characteristics. The outcome variable, reporting three or more depressive symptoms, was considered to be common (>10%); therefore, modified Poisson regression with robust standard errors was used to estimate prevalence ratios (PRs) and corresponding 95% confidence intervals (CIs) for the relationship between allostatic load group and major depressive symptoms (McNutt et al., 2003; Thorpe et al., 2017; Zou, 2004). Three models were specified: Model 1 examined the association between high allostatic load and major depressive symptoms adjusted for age; Model 2 examined the relationship between high allostatic load and major depressive symptoms adjusted for age, education, and income; and Model 3 examined the association between high allostatic load and major depressive symptoms adjusted for age education, income, smoking status, and drinking status. The appropriate weights and design factors were invoked in all of the analyses to account for the sampling design of HRS. *p* values less than .05 were considered statistically significant. All of the analyses were performed using STATA version 14 (StataCorp LP, College Station, TX).

## Results

The distribution of select characteristics for 850 black men who completed the HRS 2010 and 2012 biomarker and anthropometric assessments by allostatic load group is given in Table 1. Those who were in the low allostatic load group were on average younger (61.8 vs. 63.8 years; *p* < .05) than those participants in the high allostatic load group. No statistical differences between the low allostatic load group and the high allostatic load group were observed for education, logged household income, smoking or drinking status, or reporting of three or more depressive symptoms.

**Table 1.** The Distribution of Select Characteristics for 850 Black Men Who Completed the Health and Retirement Survey 2010 and 2012 Biomarker and Anthropometric Assessments by Allostatic Load Group

Characteristics	Low allostatic load (*n* = 629)	High allostatic load (*n* = 221)
Age (years), *m* ± *SE*	61.8 ± 0.4	63.8 ± 0.6*
Socioeconomic status		
Education level, %		
Less than high school	24.0	26.7
High school/GED	31.3	34.4
Some college	27.0	25.9
College degree	17.5	12.8
Logged household income	10.3 ± 0.1	10.2 ± 0.1
Health behaviors		
Current smoker, %	27.0	22.6
Number of drinks per week, *m* ± *SE*	1.0 ± 0.1	1.0 ± 0.1
Major depressive symptoms, %	20.0	30.0

*p < .05.

The association between allostatic load group and depressive symptoms among black men is given in Table 2. After adjusting for age, in Model 1, men who were in the high allostatic load group had a statistically significant higher prevalence of depressive symptoms (PR = 1.62, 95% CI: 1.18–2.22) than those men who were in the low allostatic load group. In Model 2, men who were in the high allostatic load group still had a statistically significant higher prevalence of major depressive symptoms (PR = 1.54, 95% CI: 1.14–2.07) than those men who were in the low allostatic load group independent of age, education, and income. In the fully adjusted model, controlling for age, education, logged household income, smoking, and drinking status, the statistically significant higher prevalence of depressive symptoms (PR = 1.61, 95% CI: 1.20–2.17) remained among men who were in the high allostatic load group relative to men who were in the low allostatic load group.

**Table 2.** Prevalence Ratios and 95% Confidence Intervals for the Association Between Allostatic Load Group and Major Depressive Symptoms Among 850 Black Men Who Completed the 2010/2012 Health and Retirement Survey Biomarker and Anthropometric Supplements

Characteristics	Model 1	Model 2	Model 3
High allostatic load group	1.62* (1.18–2.22)	1.54* (1.14–2.07)	1.61* (1.20–2.17)
Age	0.95* (0.94–0.97)	0.95* (0.93–0.97)	0.96* (0.94–0.98)
High school/GED		0.86 (0.61–1.20)	0.91 (0.65–1.27)
Some college		0.76 (0.49–1.17)	0.81 (0.53–1.25)
College graduate		0.29* (0.15–0.56)	0.36* (0.19–0.70)
Logged household income		0.91* (0.87–0.95)	0.93* (0.89–0.97)
Current smoker			1.90* (1.37–2.62)
Number of alcoholic drinks consumed per week			0.98 (0.91–1.04)

*p < .05.

## Discussion

In this study, we sought to examine the association between stress as measured by allostatic load and depressive symptoms among middle- to old age black men. Consistent with prior literature (Bey et al., 2018), we found that there was a significant association between high allostatic load and depressive symptoms among black men, independent of age, education, income, and health behaviors. A unique contribution of this study was that within-race analyses demonstrated a significant relationship between allostatic load and depressive symptoms among middle- to old age black men while Bey et al. examined the relationship among black and white adults aged 18–64. Findings from both studies indicate there is a link between depressive symptoms and high allostatic load among black men. However, our work demonstrates the physiological adaptation to stress may contribute to the reporting of three or more depressive symptoms among middle- to old age black men.

This study provides further evidence of the existence of *environmental affordances* proposed by Jackson et al. (2010). In this population of black men, the unhealthy behavior of smoking may have been used as a socially acceptable resource to cope with their stress. Over the life course, the exposure to the stress of social disadvantage along with smoking as a coping strategy likely increased their allostatic load score. For example, black men who receive poor educational experiences as adolescents may find it difficult to obtain a college degree as an adult and ultimately employment as an older adult (Bruce et al., 1998). This sequence of events exposes these black men to multiple stressors or exposure to the same stressors repeatedly over time. They may then resort to smoking to relieve the stressors associated with the effects of low educational attainment. This phenomenon makes it difficult to develop and/or maintain adaptive coping leading to allostatic load and depressive symptoms (Geronimus et al., 2006; Slopen et al., 2018; Williams & Mohammed, 2009).

The existing body of literature that has examined the relationship between stress and depressive symptoms consistently indicates that there is a relationship between social environment, discrimination, and experience of chronic stress (Archibald, 2017; Archibald et al., 2018; Hammond, 2012; Lazarus & Folkman, 1984). Research has documented that discrimination associated with racism plays an important role in depressive symptoms in black men (Mereish et al., 2016). Evidence from previous studies suggests that the link between allostatic load and depressive symptoms is likely grounded in black men’s lived experiences with the discrimination associated with racism that exposes black men to social disadvantage and social inequity (Thorpe et al., 2019, 2020). These persistent exposures to discrimination place black men in direct contact with chronic stress related to neighborhood conditions (e.g., perceptions of safety, trust, and connectedness as well as percentage of residents in poverty), working conditions, education, income, and wealth—that extends through their life course (Hudson et al., 2016; Thorpe et al., 2019; Watkins, 2012). This is important because discrimination has the potential to result in elevation of primary stress mediators such as heart rate and secondary outcomes such as blood pressure, HBA1c, waist circumference, and C-reactive protein, leading to depression (Mauss et al., 2015; McEwen et al., 2012). The evidence from this study suggests that black men’s exposure to cumulative stress and disadvantage over the life course could fuel the observed association between depressive symptoms and high allostatic load among middle- to old age black men.

There are aspects of this study that warrant comment. Because this is a cross-sectional study, inferences cannot be made regarding causality between allostatic load and depressive symptoms. A prospective study is required to understand how allostatic load influences the development of three or more depressive symptoms over time. Additional studies should also consider other types of stressors that might affect both allostatic load and depressive symptoms. One example is adverse childhood experiences (ACEs). To date, we know of no nationally representative study of older adults that contains measures of ACEs, allostatic load, load, and depressive symptoms among black men in mid- to late life. Given that theory suggests that stress exposure among black men may vary by age and periods (Jackson et al., 2010), research on this topic should examine whether coming of age during Jim Crow and the Civil Rights Movement influence the relation between ACEs and the reporting of three or more depressive symptoms.

Nevertheless, this study has a number of strengths. First, our study utilizes data from a subsample of middle- to old age black men from the HRS—a nationally representative survey of middle- to old age adults that contains biomarker assessments, psychosocial measures, and indicators of depression. The wide array of measures in the HRS represents an improvement over prior work. Furthermore, this study consisted of 850 black men with complete psychosociological data. To the best of our knowledge, we are unaware of another data source with a sufficient number of black men in mid- to late life with such rich data.

## Conclusions

Black men experience extremely high levels of stress from unfavorable social and economic circumstances. These circumstances often include black men living in segregated areas that are plagued with high stress exposure, limited availability of medical resources, poor housing conditions, high poverty, and high crime (Thorpe, Roland et al., 2013; Thorpe et al., 2020). However, stress has only been recently implicated as a major determinant in black men’s health in general and only a few studies have examined stress and depressive symptoms among middle- to old age black men. Our findings underscore the importance of extending mental health services, and fostering of protective coping strategies, or strengthening resilience for middle- to old age black men. Key stakeholders should consider developing policy that fosters environments for middle- to old age black men to seek care for mental health particularly depressive symptoms.
